# Lipid nanoparticle-mediated transcription factor EB mRNA delivery for the treatment of metabolic dysfunction-associated steatotic liver disease

**DOI:** 10.1186/s43556-026-00615-0

**Published:** 2026-10-21

**Authors:** Wenhao Ju, Chi Zhang, Tingting Zhang, Yingjie Liu, Xingyun Wang, Linjun Zeng, Yangpo Cao, Haihuan Lin, Ouhui Li, Peng George Wang, Yunjiao He, Haocheng Lu

**Affiliations:** 1 State Key Laboratory of Cardiovascular Disease, Fuwai Hospital, National Center for Cardiovascular Diseases, Key Laboratory of Innovative Cardiovascular Devices, Chinese Academy of Medical Sciences and Peking Union Medical College, Beijing, 100037 China; 2 Yunnan Key Laboratory of Innovative Diagnosis and Treatment of Circulatory Diseases, Fuwai Yunnan Hospital, Chinese Academy of Medical Sciences, Affiliated Cardiovascular Hospital of Kunming Medical University, Kunming, China; 3 Department of Pharmacology, Joint Laboratory of Guangdong-Hong Kong Universities for Vascular Homeostasis and Diseases, School of Medicine, Southern University of Science and Technology, Shenzhen, Guangdong 518055 China; 4 SUSTech Homeostatic Medicine Institute, School of Medicine, Southern University of Science and Technology, Shenzhen, 518055 China; 5 Department of Cardiology, The Eighth Affiliated Hospital, Zhongshan School of Medicine, Sun Yat-Sen University, Shenzhen, Guangdong 518033 China; 6 Department of Pharmacology, School of Medicine, Southern University of Science and Technology, Shenzhen, Guangdong 518055 China; 7 Key Laboratory of Tropical Translational Medicine of Ministry of Education, Department of Biochemistry and Molecular Biology, School of Basic Medicine and Life Sciences, Hainan Medical University, Haikou, China

**Keywords:** Metabolic dysfunction-associated steatotic liver disease, Lipid nanoparticles, Transcription factor EB, Autophagy, FGF21

## Abstract

Metabolic dysfunction-associated steatotic liver disease (MASLD) is a prevalent disorder characterized by excessive hepatic lipid accumulation, frequently progressing to steatohepatitis, fibrosis, and cirrhosis. Transcription factor EB (TFEB) coordinates lysosomal biogenesis, autophagy, and lipid catabolism, and therefore represents a potential therapeutic target; however, safe and direct strategies for selectively enhancing hepatic TFEB activity remain limited. Here, liver-targeted lipid nanoparticles (LNPs) were developed to deliver mRNAs encoding wild-type TFEB or the constitutively active TFEB S211A mutant, and their efficacy was evaluated in cellular and choline-deficient, L-amino acid-defined high-fat diet (CDAHFD)-induced mouse models of MASLD. LNP-mediated delivery increased hepatic TFEB expression, enhanced autophagic flux, and reprogrammed metabolic pathways toward fatty acid oxidation (e.g., *CPT1A* and *ACOX1*) while suppressing the expression of lipogenic genes (e.g., *FASN*, *ACC*, and *SREBP1*). Consequently, targeted TFEB mRNA therapy significantly reduced hepatic triglyceride content, attenuated steatosis, lowered MASLD activity scores, and decreased fibrotic remodeling. Mechanistically, transcriptomic profiling, ChIP-qPCR, promoter-reporter assays, and targeted knockdown models indicated that TFEB exerts its protective effects against lipid accumulation at least partly by transcriptionally activating the fibroblast growth factor 21 (FGF21) signaling pathway. Collectively, these findings establish LNP-mediated TFEB mRNA delivery as a useful preclinical approach for investigating hepatic biology and provide a targeted, transient, and non-integrating genetic strategy with potential for the treatment of MASLD.

**Supplementary Information:**

The online version contains supplementary material available at https://doi.org/10.1186/s43556-026-00615-0.

## Introduction

Metabolic dysfunction-associated steatotic liver disease (MASLD) is a complex pathological syndrome characterized by excessive hepatic lipid accumulation (> 5% of hepatocytes) in the absence of excessive alcohol consumption or other secondary causes of hepatic steatosis. Affecting approximately 25% of the global population, MASLD has emerged as a leading cause of cirrhosis and liver transplantation [1]. The disease encompasses a progressive spectrum ranging from simple steatosis to metabolic dysfunction-associated steatohepatitis (MASH), cirrhosis, and, ultimately, hepatocellular carcinoma [2]. Closely associated with metabolic syndrome, obesity, and diabetes mellitus, MASLD substantially increases the risks of cardiovascular disease and all-cause mortality [3], thereby imposing a considerable global public health burden.

The pathogenesis of MASLD is multifactorial and involves interactions between environmental factors, such as overnutrition, and genetic susceptibility, including polymorphisms in *PNPLA3*, *TM6SF2*, and *HSD17B13* [4, 5]. A central feature of disease progression is excessive hepatocellular lipid accumulation, which induces lipotoxicity, aggravates systemic insulin resistance [6], and promotes hepatic inflammation and fibrosis. Despite this growing clinical burden, pharmacological interventions remain limited [7]. While recent advances, including selective thyroid hormone receptor-beta (THR-β) agonists, have shown promise for the treatment of MASH [8], other therapeutic candidates, including PPAR agonists, GLP-1 receptor agonists [9], and FGF analogs [10], remain under clinical evaluation. Thus, there remains a substantial need for novel and highly targeted therapeutic strategies.

Transcription factor EB (TFEB), a master transcriptional regulator of the MiTF/TFE family, represents a compelling target for correcting the metabolic dysregulation underlying MASLD [11]. Under conditions of metabolic stress or nutrient deprivation, TFEB translocates to the nucleus to drive the expression of genes governing lysosomal biogenesis [12], autophagy [13], and lipid metabolism [14]. In human liver biopsies, hepatic TFEB activity has been reported to correlate inversely with the severity of steatosis. Preclinical models further demonstrate that TFEB overexpression effectively attenuates high-fat diet-induced hepatic lipid accumulation, suggesting that impaired TFEB-mediated lysosomal and autophagic function contributes to MASLD progression [15].

Although activating TFEB has shown potential to ameliorate MASLD, current pharmacological approaches face significant translational hurdles. Most pharmacological TFEB activators act indirectly by inhibiting mTORC1 [16–18]. Because mTORC1 is ubiquitously expressed and regulates essential cellular processes across multiple tissues, its systemic inhibition can cause adverse effects such as immunosuppression, hyperlipidemia, and hyperglycemia. These off-target systemic toxicities substantially limit the clinical translation of small-molecule TFEB activators [19].

To overcome the limitations of systemic mTORC1 inhibition, a direct, liver-targeted genetic strategy involving TFEB mRNA delivery using lipid nanoparticles (LNPs) was investigated. LNPs were clinically validated as delivery platforms during the COVID-19 pandemic [20]. Following systemic administration, LNPs preferentially accumulate in the liver [21], owing in part to the fenestrated hepatic endothelium and the formation of an apolipoprotein E-enriched protein corona that facilitates hepatocyte uptake [22]. This inherent liver tropism makes LNPs attractive delivery vehicles for the treatment of hepatic diseases. Compared with viral gene therapy, LNP-mediated mRNA delivery is transient and non-integrating, thereby reducing the risks associated with insertional mutagenesis and persistent transgene expression [22, 23]. This study investigated whether LNP-mediated delivery of TFEB mRNA enhances hepatic autophagy and lipid catabolism, thereby attenuating MASLD-associated liver injury in preclinical models.

## Results

### TFEB is dynamically dysregulated during MASLD progression

**Fig. 1:**
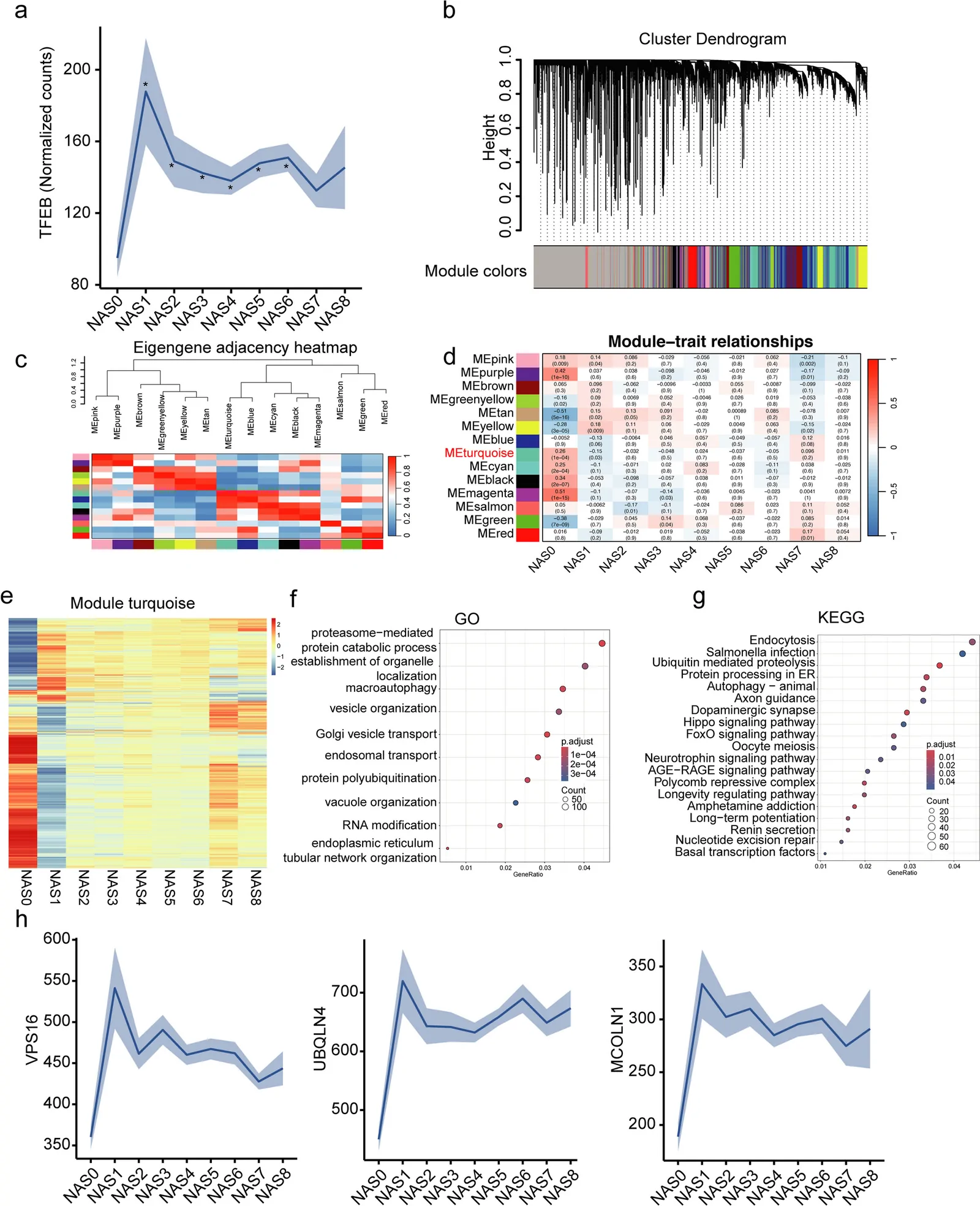
WGCNA analysis of MASLD-related genes. **a** TFEB expression level in different stages of MASLD biopsy samples. Data showing DESeq2 normalized counts, presented as mean ± SEM. * *P* < 0.05 compared with NAS 0 group. **b** Clustering dendrograms showing hierarchical clustering of modules identified by WGCNA. **c** Hierarchical clustering and heatmap of eigengene adjacencies in the consensus eigengene network. **d** Heatmap showing correlation of the corresponding module eigengene (row) with the NAS (column). **e** Heatmap showing expression of individual genes in the turquoise module. Color indicates calculated z score. **f**-**g** GO (BP) and KEGG pathway enrichment of genes in the turquoise module. **h** Gene expression levels in different stages of MASLD biopsy samples. Data are DESeq2-normalized counts and are presented as mean ± SEM

To investigate the therapeutic potential of TFEB in human MASLD, changes in TFEB expression across NAFLD progression were evaluated using a publicly available RNA-sequencing dataset comprising 216 liver biopsy samples with different nonalcoholic fatty liver disease activity scores (NAS; GSE135251) [24]. Analysis of this transcriptomic dataset spanning the full spectrum of MASLD revealed dynamic changes in TFEB gene expression from control liver samples (NAS 0) to samples with late-stage MASLD (NAS 8). TFEB expression followed a biphasic pattern, increasing at NAS 1, declining through intermediate stages, and increasing modestly again at NAS 8 (Fig. 1a). Weighted gene co-expression network analysis (WGCNA) [25] was then performed to identify genes whose expression patterns paralleled that of TFEB during MASLD progression in humans (Fig. 1b). Network topology was analyzed using a soft-thresholding power of 12. Fourteen distinct modules with different expression patterns across MASLD stages were identified (Fig. 1c, d). TFEB clustered within the turquoise module. Module-trait relationship analysis showed that this module was positively associated with both the healthy control phenotype (NAS 0) and advanced MASLD stage (NAS 7–8) (Fig. 1d). Heatmap visualization confirmed that genes in the turquoise module followed an expression trajectory similar to that of TFEB (Fig. 1e). These dynamic expression patterns suggested that TFEB and other genes within this module may have distinct roles during the early and late stages of MASLD. Gene Ontology (GO) and Kyoto Encyclopedia of Genes and Genomes (KEGG) enrichment analyses of the turquoise module identified autophagy-related processes, including macroautophagy, vesicle trafficking, and endosomal transport (Fig. 1f, g). These processes are regulated downstream of TFEB [12]. Representative TFEB-regulated autophagy genes, including VPS16, UBQLN4, and MCOLN1, exhibited expression patterns that paralleled that of TFEB across disease stages. These findings indicate that TFEB and its downstream autophagy-related targets undergo dynamic regulation during MASLD progression and warrant further investigation as potential therapeutic targets (Fig. 1h).

### LNPs enable liver-targeted delivery of TFEB mRNA

**Fig. 2:**
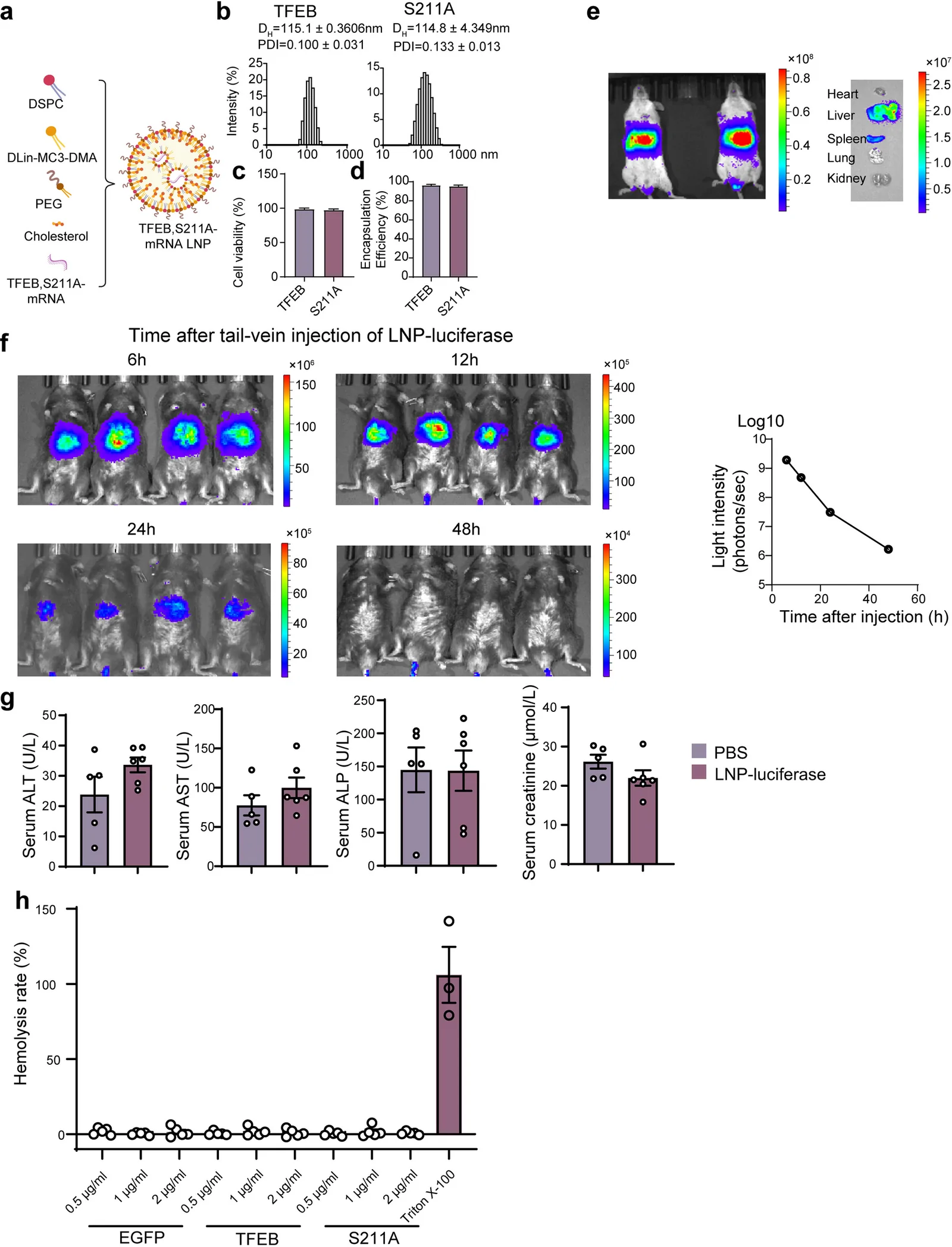
Preparation and characterization of lipid nanoparticles (LNPs). **a** Schematic of the preparation of LNPs encapsulating TFEB or S211A mRNA. **b** Particle size distribution of LNPs. **c** Cytotoxicity of LNP was determined by CCK-8 assay. **d** Encapsulation Efficiency of LNP. **e** Detection of organ distribution of LNP by in vivo luciferase imaging. Mice were injected with 10 μg of luciferase-mRNA encapsulated in LNP via tail vein, followed by an intraperitoneal injection of luciferin 6 h later. Luminescence signals were detected using a small animal in vivo imaging system. **f** Luminescence signals 6 h, 12 h, 24 h, and 48 h after LNP-luciferase tail-vein injection. **g** Serum ALT, AST, ALP, and creatinine were measured 24 h after tail-vein injection of 50 μg LNP-luciferase. **h** In vitro hemolysis assay to assess LNP biocompatibility. Data were expressed as mean ± SEM

LNPs encapsulating mRNA encoding either wild-type TFEB or TFEB-S211A were formulated using DSPC, DLin-MC3-DMA, PEG, cholesterol, and the corresponding mRNA in a microfluidic device (Fig. 2a). Substitution of Ser211 with alanine renders TFEB constitutively active by preventing phosphorylation at this site. The TFEB-S211A mutant was therefore included to determine whether constitutive TFEB activation produced a greater therapeutic effect. The mean particle sizes of LNP-TFEB and LNP-S211A were approximately 114–115 nm (Fig. 2b). A CCK-8 assay showed that both LNP-TFEB and LNP-S211A exhibited negligible cytotoxicity (Fig. 2c). The encapsulation efficiencies of LNP-TFEB and LNP-S211A were both nearly 100% (Fig. 2d). An LNP formulation encapsulating luciferase mRNA (LNP-luciferase) was used to assess tissue distribution after tail-vein injection. Luciferase imaging in mice demonstrated strong hepatic targeting by the LNPs (Fig. 2e), supporting their suitability for liver-directed delivery.

To assess the duration of luciferase expression in vivo, LNP-luciferase was administered to mice via tail-vein injection. At 6, 12, 24, and 48 h after injection, luciferin was administered intraperitoneally, followed by in vivo bioluminescence imaging to measure signal intensity. The luminescence signal peaked at 6 h and was nearly undetectable at 48 h (Fig. 2f). To evaluate the acute toxicity of the LNPs in vivo, serum was collected from mice 24 h after injection of 50 µg of LNP-luciferase. Serum levels of alanine transaminase (ALT), aspartate transaminase (AST), alkaline phosphatase (ALP), and creatinine were not significantly altered, indicating that LNP treatment caused no detectable acute hepatotoxicity or nephrotoxicity (Fig. 2g). Biocompatibility was also evaluated using a hemolysis assay. LNP treatment resulted in less than 5% red blood cell lysis (Fig. 2h).

### LNP-TFEB promotes autophagy and reduces lipid accumulation in Huh-7 cells

**Fig. 3:**
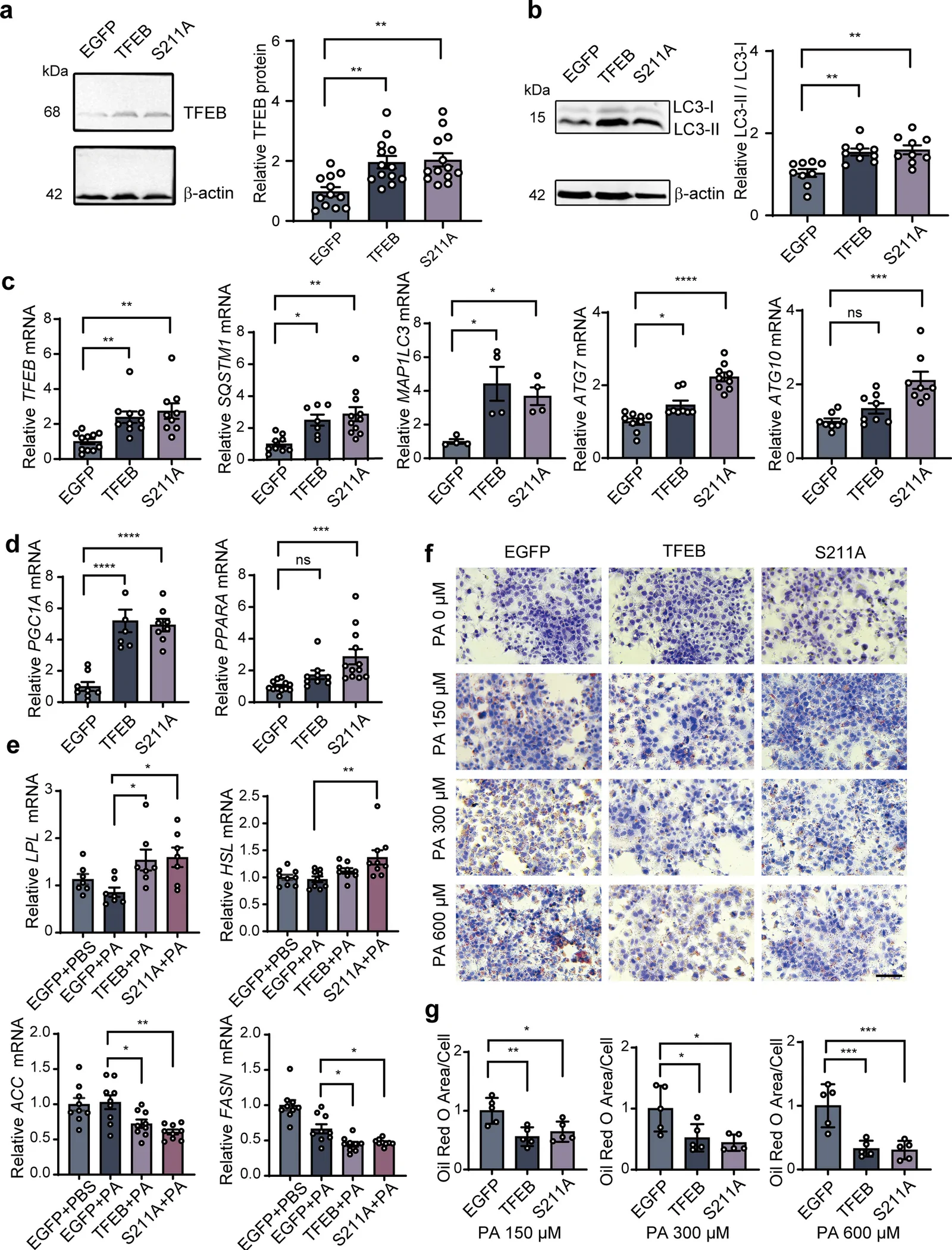
LNP-TFEB promoted TFEB expression in Huh-7 cells. **a**-**d** Huh-7 cells were treated with LNP-EGFP, LNP-TFEB, or LNP-S211A at 1 µg/ml for 24 h. **a** TFEB expression was assessed by Western blotting (*n* = 12 per group). **b** LC3-II/LC3-I was assessed using Western blot (*n* = 9 per group). **c** The expression of autophagy-related genes was determined by qPCR (*n* = 4 or 8 per group). **d** The expression of genes related to lipid synthesis and lipid metabolism was determined by qPCR. **e**–**g** Huh-7 cells were treated with LNP-EGFP, TFEB, or S211A at 1 µg/ml for 6 h, then PA was added at concentrations of 0 µM, 150 µM, 300 µM, and 600 µM, and incubation was continued for 18 h. In the qPCR experiment, PA was added at a concentration of 300 µM. **e** The expression of genes related to lipid metabolism and lipid synthesis was determined by qPCR (*n* = 6 or 9 per group). **f**-**g** Oil Red O was used to quantify the lipid content in the cells (*n* = 5 per group). Scale bar = 100 μm. Data were expressed as mean ± SEM. *P < 0.05, **P < 0.01, ***P < 0.001, ****P < 0.0001

The capacity of LNP-mediated mRNA delivery to increase TFEB expression in Huh-7 cells was evaluated using mRNA encoding wild-type TFEB or its S211A mutant (hereafter S211A), a constitutively active form of TFEB. Huh-7 cells were treated with LNP-TFEB or LNP-S211A at 1 µg/mL, with LNP-EGFP serving as the negative control. After 24 h of treatment, both LNP-TFEB and LNP-S211A significantly increased TFEB expression in Huh-7 cells (Fig. 3a) and elevated the LC3-II/LC3-I ratio, consistent with altered autophagy-related signaling (Fig. 3b). qPCR analysis showed that both formulations increased the expression of autophagy-related genes, including *SQSTM1/P62, MAP1LC3, ATG7, and ATG10* (Fig. 3c), as well as lipid metabolism-related genes, including *PPARGC1A* and *PPARA* (Fig. 3d). The effects of TFEB overexpression on cellular lipid metabolism were subsequently evaluated using palmitic acid (PA) to induce lipid accumulation. Huh-7 cells were treated with LNP-EGFP, LNP-TFEB, or LNP-S211A for 6 h and then incubated with 300 μM PA for 18 h. qPCR analysis showed that both LNP-TFEB and LNP-S211A significantly downregulated the expression of genes involved in lipid synthesis, including *ACC* and *FASN*, while upregulating genes involved in lipid catabolism, including *LPL* and *HSL* (Fig. 3e). Oil Red O staining of Huh-7 cells also demonstrated that both LNP-TFEB and LNP-S211A significantly reduced intracellular lipid accumulation (Fig. 3f, g).

### LNP-TFEB attenuates CDAHFD-induced MASLD in mice

**Fig. 4:**
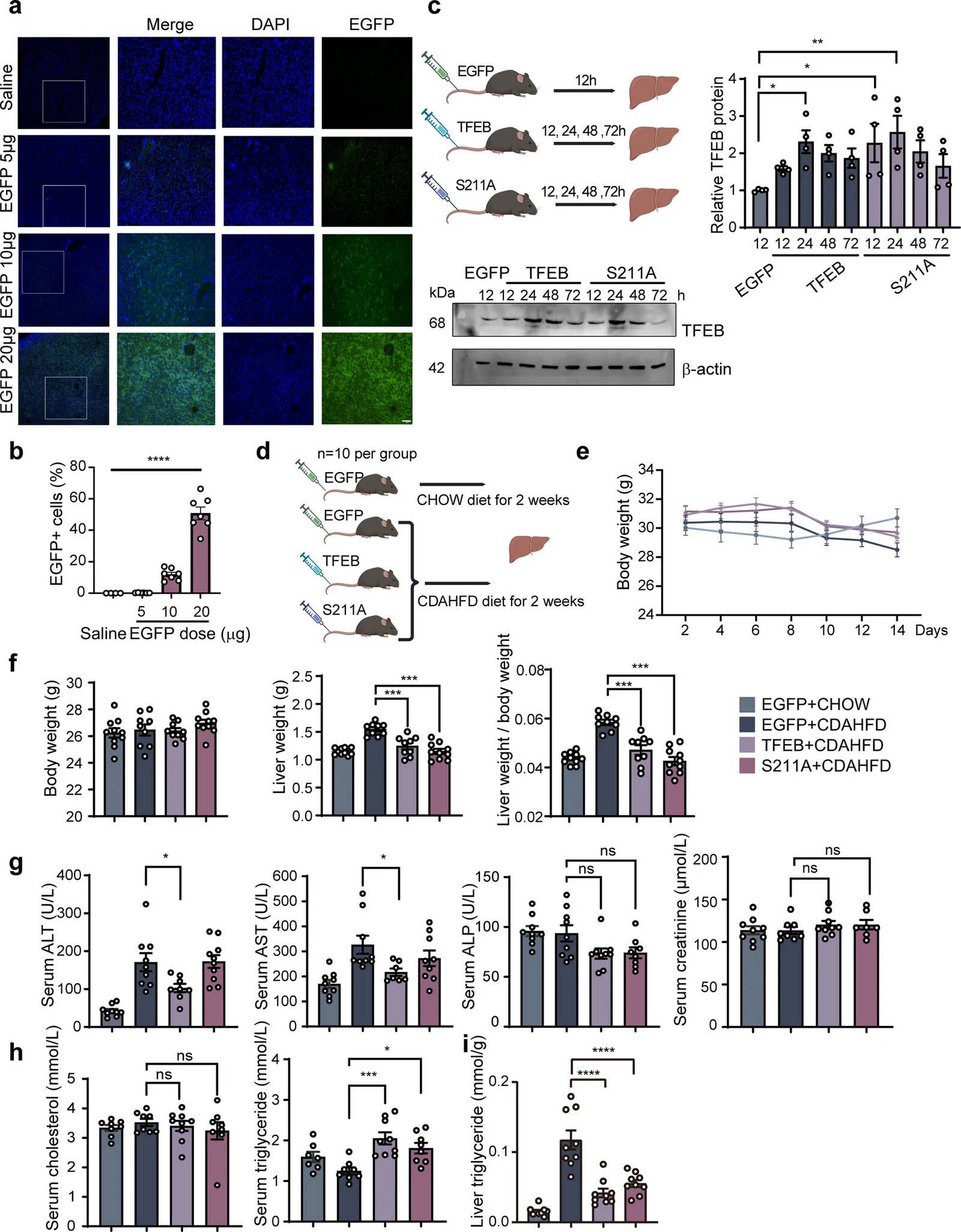
LNP-TFEB attenuated MASLD in CDAHFD-fed mice. **a**-**b** LNP-EGFP (5 µg, 10 µg, 20 µg) or negative control (saline) was administered via tail vein injection. After 24 h, liver sections were collected for fluorescence imaging (*n* = 6 or 7 per group). Scale bar = 100 μm. **c** LNP-EGFP, TFEB or S211A (20 μg per mouse) were administered via tail vein injection. Liver proteins were collected 12 h, 24 h, 48 h, and 72 h post-injection and analyzed for TFEB expression using Western blot (*n* = 4 per group). **d** Mice were fed a chow or CDAHFD diet continuously for 2 weeks. LNP-EGFP, LNP-TFEB, or LNP-S211A (20 µg) was administered by tail-vein injection every 2 days (*n* = 10 per group). **e** Body weight was determined each time before LNP administration. **f**-**i** After 2 weeks, the mice were euthanized. **f** Body weight and liver weight and liver index (liver weight/body weight ratio) were measured. **g**-**h** Serum ALT, AST, and ALP activities and creatinine, cholesterol, and triglyceride concentrations were measured using a biochemical analyzer. **i** Liver triglyceride content was determined using a TG content assay kit. Data were expressed as mean ± SEM. *P < 0.05, **P < 0.01, ***P < 0.001, ****P < 0.0001

To determine an optimal in vivo dose, mice received LNP-EGFP at doses of 5, 10, or 20 μg via tail-vein injection, with saline serving as the negative control. EGFP fluorescence in liver sections was assessed 24 h later by fluorescence microscopy and normalized to DAPI staining. The 20-μg dose yielded approximately 60% EGFP-positive liver cells (Fig. 4a, b) and was therefore selected to evaluate hepatic TFEB delivery without unnecessary dose escalation. Higher doses of TFEB or S211A mRNA were not tested in the MASLD model, although 50 μg of LNP-luciferase did not produce acute biochemical evidence of hepatotoxicity or nephrotoxicity (Fig. 2g). Because both mRNA and the translated TFEB protein degrade over time in vivo, the dosing interval was subsequently determined. After tail-vein administration of 20 μg of LNP-TFEB or LNP-S211A, hepatic TFEB protein levels peaked at 24 h, decreased at 48 h, and returned by 72 h to levels comparable to those in LNP-EGFP-treated control mice (Fig. 4c). Therefore, LNPs were administered every 2 days in the subsequent MASLD experiments. A mouse model of MASLD was established by feeding mice a choline-deficient, L-amino acid-defined, high-fat diet (CDAHFD) for 2 weeks while simultaneously administering LNP-EGFP, LNP-TFEB, or LNP-S211A (Fig. 4d). Chow-fed mice treated with LNP-EGFP served as controls. Body weight was monitored before each LNP administration, and no overall differences were observed among the four groups (Fig. 4e, f). Compared with chow-fed controls, CDAHFD significantly increased liver weight and the liver index, defined as the liver weight/body weight ratio, indicating successful induction of MASLD (Fig. 4f). Treatment with LNP-TFEB or LNP-S211A did not affect mTORC1 activity in vivo (Fig. S1), suggesting that the observed effects did not require detectable suppression of mTORC1 activity. Both LNP-TFEB and LNP-S211A attenuated the CDAHFD-induced increase in liver weight (Fig. 4f). Serum ALT and AST levels were lower in the LNP-TFEB group, suggesting reduced hepatic injury in mice with MASLD. Serum creatinine was comparable among groups, indicating no detectable nephrotoxicity associated with LNP treatment (Fig. 4g). Serum total cholesterol levels were similar across the groups, whereas LNP-TFEB and LNP-S211A increased serum triglyceride levels and reduced hepatic triglyceride content (Fig. 4h, i). This reciprocal pattern may reflect enhanced triglyceride export from the liver, although direct flux studies will be required to confirm this interpretation. Histological analysis of H&E-stained sections showed that LNP-TFEB and LNP-S211A did not cause detectable damage to the heart, kidneys, lungs, spleen, or adipose tissue (Fig. S2). Notably, LNP-S211A reduced hepatic triglyceride content to an extent similar to that achieved with LNP-TFEB but did not reduce serum AST or ALT levels, suggesting that excessive or constitutive TFEB activation may have a narrower safety window.

### LNP-TFEB and S211A enhance hepatic autophagy and lipid metabolic remodeling

**Fig. 5:**
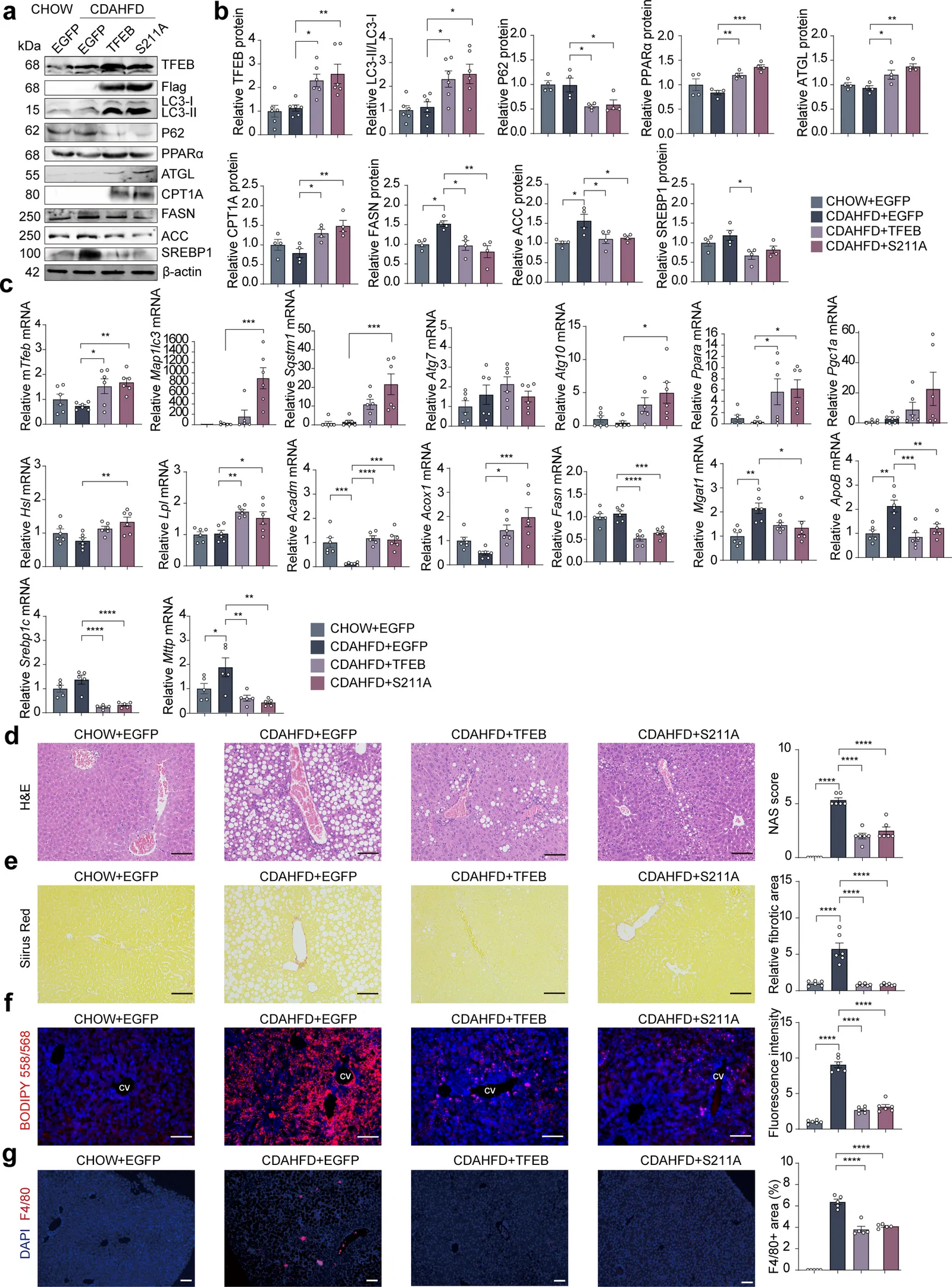
LNP-TFEB and S211A enhanced autophagy and promoted lipid metabolism in the mouse liver. **a** Representative Western blot images and **b** corresponding quantitative analysis of TFEB (TFEB and FLAG-tag detection confirmed LNP-mediated expression of TFEB and S211A), autophagy-related proteins (LC3, P62), lipid metabolism-related proteins (PPARα, ATGL, CPT1A) and lipid synthesis-related proteins (FASN, ACC, SREBP1) in mouse liver (*n* = 4 or 6 per group). **c** Relative hepatic mRNA expression levels of autophagy-related genes, lipid catabolism-related genes and lipid synthesis and transport-related genes, as determined by qPCR (*n* = 5 or 6 per group). **d** Representative hematoxylin and eosin (H&E) staining images of liver sections and the corresponding NAFLD activity score (NAS) (*n* = 6 per group). **e** Representative images of Sirius Red staining and quantification of the relative fibrotic area (*n* = 6 per group). **f** Representative BODIPY fluorescence staining images of neutral lipids in liver sections and quantification of fluorescence intensity. cv: Central vein (*n* = 6 per group). **g** Representative F4/80 immunofluorescence staining images showing macrophage infiltration and the corresponding quantitative analysis (*n* = 5 per group). Scale bar = 100 μm. Data are expressed as mean ± SEM. *P < 0.05, **P < 0.01, ***P < 0.001, ****P < 0.0001

Previous studies have established TFEB as a pivotal regulator of hepatic autophagy and lipid catabolism [26]. To characterize the molecular alterations induced by targeted treatment, hepatic protein expression profiles were compared across the experimental groups by Western blotting. LNP-TFEB and LNP-S211A significantly increased TFEB protein levels in the mouse liver, and LNP-mediated overexpression of TFEB and S211A was confirmed by FLAG-tag detection (Fig. 5a). Both treatments increased the LC3-II/LC3-I ratio and decreased p62 protein levels, consistent with altered autophagy-related activity (Fig. 5a). Concurrently, the protein levels of key enzymes involved in lipid catabolism, including PPARα, ATGL, and CPT1A, were markedly increased, whereas those of the lipogenic regulators FASN, ACC, and SREBP1 were substantially decreased (Fig. 5a, b). At the transcriptional level, treatment significantly increased the mRNA levels of autophagy-related genes, particularly *Map1lc3* and *Sqstm1*, with moderate increases in *Atg7* and *Atg10* (Fig. 5c). Furthermore, the expression of genes driving lipid oxidation and lipolysis (e.g., *Ppara*, *Ppargc1a*, *Hsl*, *Lpl*, *Acadm*, *Acox1*) was broadly enhanced (Fig. 5c), while genes orchestrating lipid biosynthesis and transport (e.g., *Fasn*, *Mgat1*, *ApoB*, *Srebp1c*, *Mttp*) were significantly repressed (Fig. 5c). Endogenous *Tfeb* mRNA (m*Tfeb*) was also modestly increased after treatment with LNP-TFEB or LNP-S211A, suggesting possible autoregulation of *Tfeb* expression.

These molecular changes were accompanied by histopathological improvements. Hematoxylin and eosin (H&E) staining, together with reduced nonalcoholic fatty liver disease activity scores (NAS), showed that LNP-TFEB and LNP-S211A mitigated hepatic steatosis (Fig. 5d). Sirius Red staining and morphometric quantification demonstrated a reduction in the relative fibrotic area in treated livers (Fig. 5e). BODIPY fluorescence staining further showed reduced neutral lipid droplet accumulation in hepatocytes (Fig. 5f). F4/80 immunofluorescence revealed reduced macrophage infiltration after treatment with LNP-TFEB or LNP-S211A, suggesting attenuation of the inflammatory microenvironment (Fig. 5g). Together, these findings indicate that LNP-mediated delivery of TFEB or its S211A variant attenuates hepatic steatosis, fibrotic changes, and macrophage infiltration in CDAHFD-fed mice.

### RNA sequencing reveals TFEB-dependent reprogramming of hepatic metabolism and inflammation

**Fig. 6:**
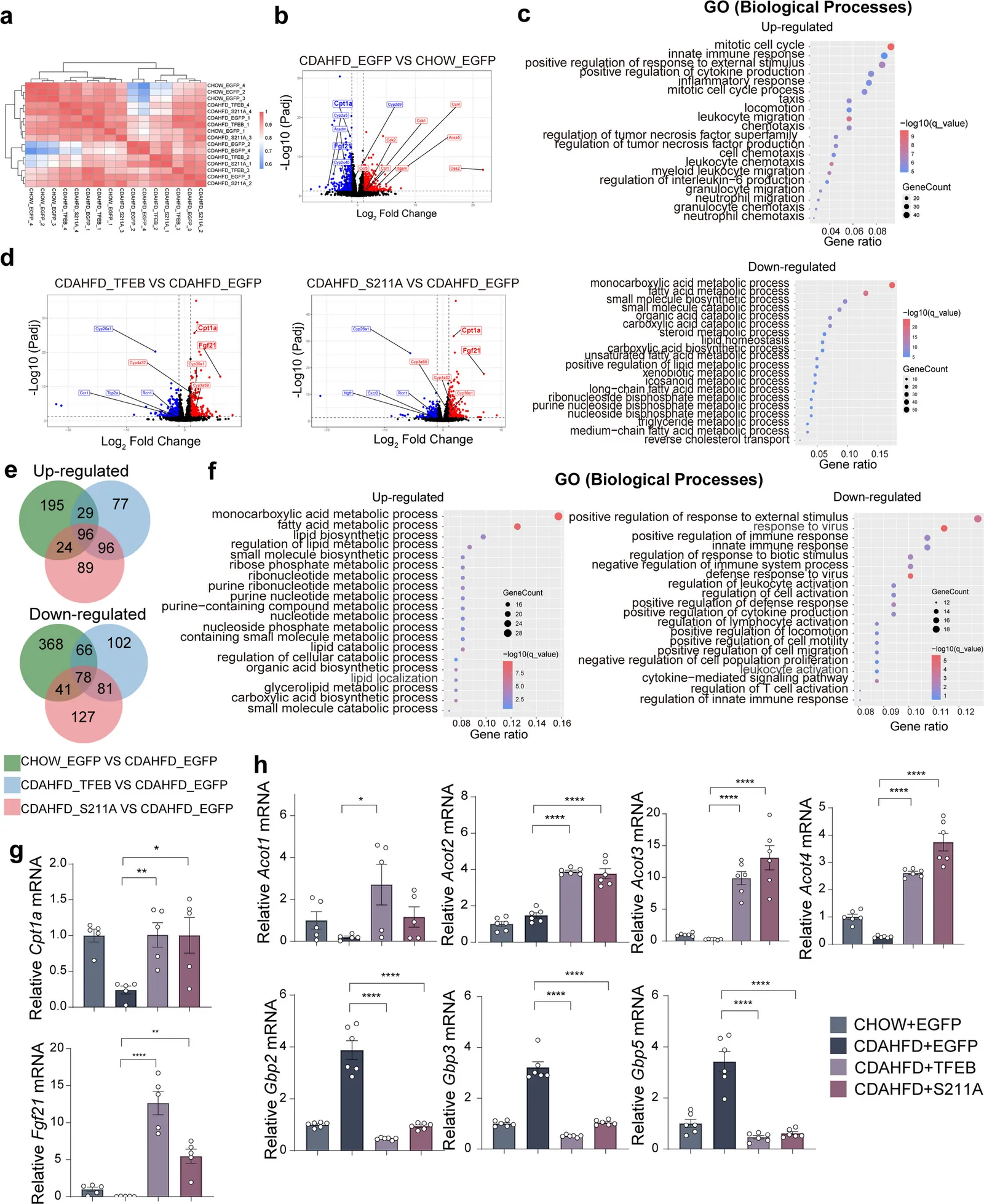
RNA sequencing reveals the impact of TFEB on the mouse liver transcriptome. **a** The heatmap shows pairwise correlations among samples. **b** The volcano plot showed the gene expression differences between CDAHFD_EGFP and CHOW_EGFP (*n* = 4 per group). **c** GO enrichment analysis of the differentially expressed genes in panel **b**. **d** The volcano plots showed the gene expression differences between CDAHFD_TFEB or CDAHFD_S211A vs CDAHFD_EGFP. **e** The Venn diagram shows the genes that are upregulated or downregulated in CDAHFD_TFEB, CDAHFD_S211A, and CHOW_EGFP compared to CDAHFD_EGFP. Among them, there are 96 commonly upregulated genes and 78 commonly downregulated genes. **f** GO enrichment analysis was performed on genes upregulated and downregulated by both LNP-TFEB and LNP-S211A. **g**-**h** The expression levels of *Cpt1a*, *Fgf21*, *Acot1-4*, *Gbp2*, *Gbp3*, and *Gbp5* in mouse liver were determined by qPCR (*n* = 5 or 6 per group). Data are expressed as mean ± SEM. *P < 0.05, **P < 0.01, ***P < 0.001, ****P < 0.0001

To characterize the transcriptional changes induced by LNP-TFEB and LNP-S211A, RNA sequencing was performed on mouse liver samples. The correlation heatmap demonstrated high correlations among samples within each group (Fig. 6a). Liver transcriptomes from CDAHFD-fed and chow-fed mice were first compared. CDAHFD feeding significantly upregulated hepatic genes associated with the cell cycle, inflammatory responses, and immune cell infiltration (e.g., *Ccr2*, *Ccr5*, and *Itgam*), while significantly downregulating genes involved in lipid catabolism (e.g., *Cpt1a* and *Fgf21*) (Fig. 6b and Table S1). Gene Ontology (GO) term enrichment showed that CDAHFD feeding enhanced inflammatory responses and suppressed fatty acid metabolism (Fig. 6c). Previous studies have shown that activation of the PPARs/CPT1A pathway promotes fatty acid β-oxidation and reduces lipid accumulation in hepatocytes [27]. FGF21 can improve glucose and lipid metabolism through the PGC1α and ERK pathways, thereby alleviating MASLD. Comparison of liver transcriptomes from CDAHFD-fed mice treated with LNP-TFEB or LNP-S211A versus LNP-EGFP showed that both TFEB and S211A reversed the CDAHFD-induced downregulation of *Cpt1a* and *Fgf21* and reduced the expression of genes associated with inflammatory responses (Fig. 6d, Tables S2 and S3). The differentially expressed genes in the Chow_EGFP, CDAHFD_TFEB, and CDAHFD_S211A groups relative to the CDAHFD_EGFP group were compared, and shared differentially expressed genes were identified using a Venn diagram (Fig. 6e). Among these shared genes, 96 were upregulated, and 78 were downregulated. These overlapping genes were dysregulated by CDAHFD feeding and reversed by both treatments. GO enrichment analysis of genes concordantly upregulated or downregulated by LNP-TFEB and LNP-S211A showed that both treatments were associated with transcriptional programs reflecting enhanced lipid metabolism and attenuated inflammatory responses, thereby counteracting the effects of CDAHFD feeding (Fig. 6f). qPCR analysis further showed that LNP-TFEB and LNP-S211A reversed the CDAHFD-induced decreases in the expression of *Cpt1a* and *Fgf21* (Fig. 6g), as well as fatty acid metabolism-related genes *Acot1*, *Acot2*, *Acot3*, and *Acot4* (Fig. 6h). In addition, both treatments suppressed the expression of interferon-induced guanylate-binding proteins, including *Gbp2*, *Gbp3*, and *Gbp5* (Fig. 6h), suggesting that TFEB may attenuate inflammatory responses in MASLD. LNP-TFEB also reduced the expression of inflammation- and fibrosis-related genes in the livers of mice with MASLD (Fig. S3a, b).

### TFEB activates FGF21 signaling during MASLD attenuation

**Fig. 7:**
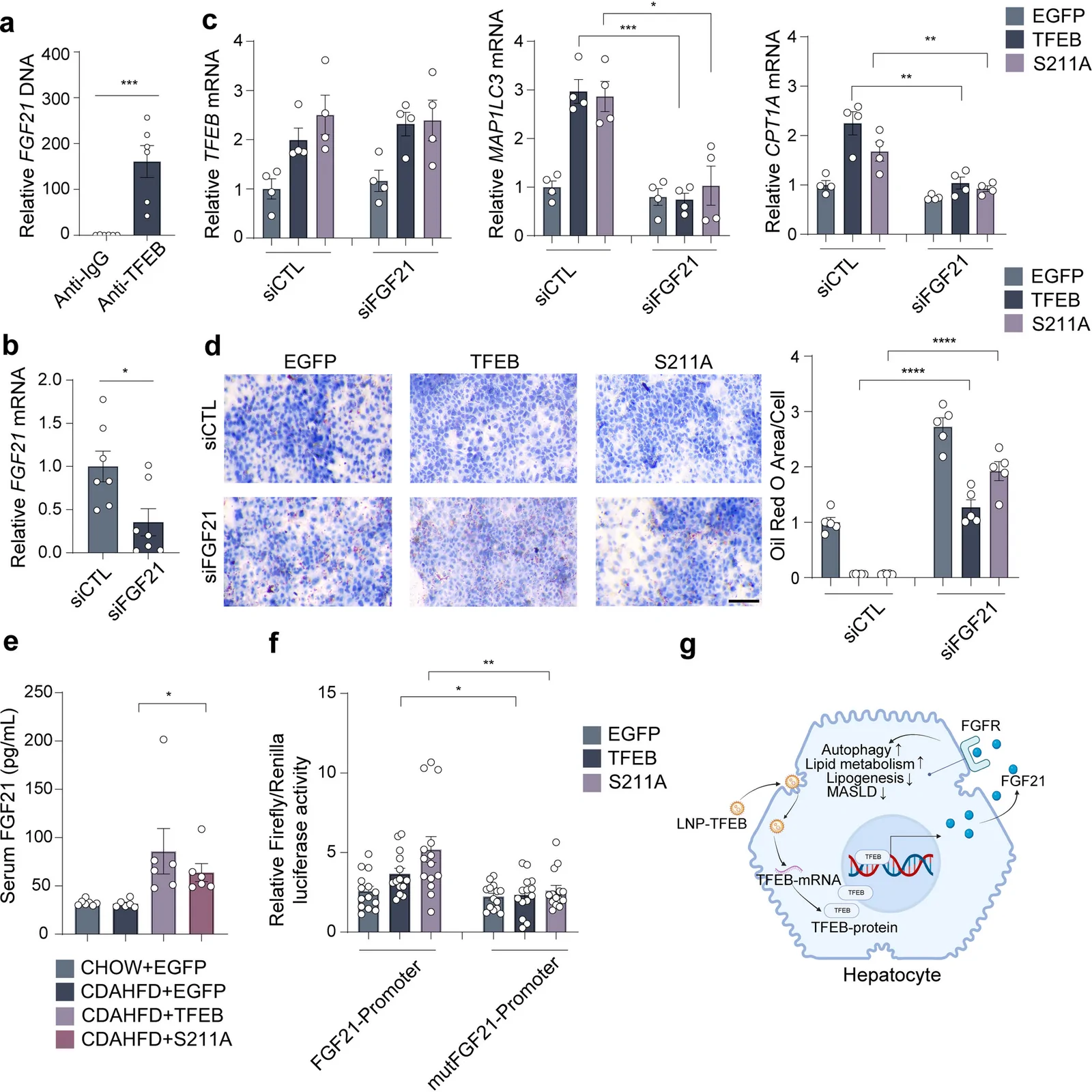
TFEB attenuates MASLD-associated phenotypes through the FGF21 pathway. **a** ChIP-qPCR assay of TFEB binding to the FGF21 promoter (*n* = 6). **b** FGF21 mRNA was determined by qPCR after siFGF21 transfection for 48 h (*n* = 7). **c** TFEB, LC3, and CPT1a mRNA were determined by qPCR after siFGF21 transfection for 48 h (*n* = 4 per group). **d** Huh-7 cells were transfected with siCTL or siFGF21. After 24 h, LNP-EGFP, LNP-TFEB, or LNP-S211A were added to cells. After 6 h, cells were treated with 300 μM PA for 24 h. Oil Red O staining was used to detect lipid accumulation in Huh-7 cells (*n* = 5 per group). Scale bar = 100 μm. **e** Serum FGF21 concentrations in the indicated mouse groups (*n* = 6 per group). **f** HEK293A cells co-transfected with a Renilla luciferase expression plasmid and either FGF21-promoter firefly luciferase or mutFGF21-promoter firefly luciferase, followed by treatment with LNP-EGFP, LNP-TFEB, or LNP-S211A. Relative luminescence intensity was calculated by Firefly/Renilla ratio (*n* = 14 per group). **g** Schematic showing how LNP-TFEB may attenuate MASLD through FGF21 signaling. Data were expressed as mean ± SEM. *P < 0.05, **P < 0.01, ***P < 0.001, ****P < 0.0001

LNP-TFEB significantly increased hepatic *Fgf21* mRNA levels. Previous studies have shown that FGF21 can prevent or ameliorate MASLD [28–31]. Therefore, whether TFEB attenuates MASLD-associated lipid accumulation partly by increasing FGF21 expression was examined. Analysis of publicly available TFEB ChIP-seq data generated in HeLa and BeWo cells (GSE260617) identified TFEB-binding peaks within the FGF21 promoter region (Fig. S4a). ChIP-qPCR confirmed that TFEB bound to a putative CLEAR box in the FGF21 promoter in Huh-7 cells (Fig. 7a and Fig. S4b). Treatment with Torin1, a canonical mTOR inhibitor and TFEB activator, also increased FGF21 expression, further supporting FGF21 as a downstream target of TFEB (Fig. S4c). To evaluate the contribution of FGF21 signaling to TFEB activity in hepatocytes, FGF21 was knocked down using siRNA in Huh-7 cells (Fig. 7b). FGF21 knockdown blunted the LNP-TFEB- and LNP-S211A-induced increases in *MAP1LC3* and *CPT1A* without directly affecting TFEB expression (Fig. 7c). Oil Red O staining showed that FGF21 knockdown diminished the ability of LNP-TFEB and LNP-S211A to reduce lipid accumulation in Huh-7 cells (Fig. 7d). Serum FGF21 levels were also increased in mice with MASLD after treatment with either LNP-TFEB or LNP-S211A (Fig. 7e). Dual-luciferase reporter assays further showed that TFEB activated the FGF21 promoter through a CLEAR-box sequence (5′-gtcacgtgat-3′) (Fig. 7f). FGF21 is also a known transcriptional target of PPARα, which was induced in the livers of LNP-TFEB-treated mice. Treatment with the PPARα antagonist GW6471 partially abrogated the effects of LNP-TFEB and LNP-S211A on lipid accumulation in Huh-7 cells (Fig. S4d). These findings suggest that the protective effects of TFEB in MASLD models are mediated, at least in part, through the transcriptional upregulation of FGF21 (Fig. 7g).

## Discussion

Several small-molecule compounds, including ajugol, nuciferine, and ezetimibe [18, 32, 33], are being investigated for their ability to activate TFEB in metabolic liver disease. These compounds generally promote TFEB dephosphorylation and nuclear translocation indirectly, often through AMPK activation or mTORC1 inhibition. Because systemic mTORC1 inhibition can disrupt hematopoiesis and whole-body metabolism, strategies that enhance hepatic TFEB activity without broadly suppressing mTORC1 may be valuable. In the present study, LNPs were used for liver-targeted delivery of TFEB mRNA. The findings position LNP-mediated TFEB delivery primarily as a preclinical tool for investigating hepatic TFEB biology in vivo and as a potential starting point for future MASLD interventions rather than as a mature therapeutic strategy.

Transcriptomic analysis indicated that TFEB overexpression reversed the MASLD-induced suppression of *Fgf21* and *Cpt1a* in murine livers. Fibroblast growth factors (FGFs) are multifunctional signaling peptides, among which FGF21 has a key endocrine role in maintaining glucose and lipid homeostasis. Previous studies have shown that FGF21 regulates TFEB activity through the AMPK–MCOLN1–calcineurin signaling pathway and that the liver is the primary source of FGF21 in both rodents and humans [31, 34]. The present findings indicate a reciprocal relationship in which hepatic TFEB overexpression increases FGF21 expression. Furthermore, CPT1A, a key enzyme involved in transporting long-chain fatty acids into mitochondria for β-oxidation, was upregulated by LNP-TFEB treatment, thereby facilitating lipid catabolism and reducing hepatic fatty acid accumulation [35, 36]. Collectively, these findings suggest that the TFEB-FGF21 axis may contribute to the attenuation of MASLD-related liver injury. However, whether TFEB activation by conventional mTOR inhibitors also depends on this FGF21 pathway remains to be determined.

Notably, whereas LNP-TFEB significantly reduced serum ALT and AST levels, the constitutively active mutant LNP-S211A failed to provide comparable protection against liver injury. This discrepancy suggests that TFEB activation may have a narrow therapeutic window. Sustained or excessive TFEB hyperactivation induced by LNP-S211A may disrupt lysosomal or autophagy-related homeostasis and paradoxically exacerbate hepatocellular injury. Consequently, the extent of autophagy activation may require careful titration, underscoring the need to calibrate TFEB expression precisely to maintain autophagic flux within a safe physiological range.

Several limitations should be acknowledged. First, the CDAHFD mouse model does not fully recapitulate the complete pathophysiological spectrum of human MASLD/MASH, particularly systemic features such as obesity and insulin resistance, thereby limiting the immediate translational relevance of these findings. Second, the treatment period was relatively short (2 weeks); therefore, the durability of the therapeutic effects after discontinuation of LNP-TFEB treatment remains unknown. Third, potential sex-related differences in MASLD progression and lipid metabolism were not fully evaluated, necessitating the inclusion of both sexes in future preclinical studies. Finally, mechanistic validation using FGF21 silencing was limited to in vitro models; in vivo validation is therefore needed to establish the contribution of this pathway.

Future studies should define the stage-specific roles of autophagy during MASLD progression. The duration of TFEB expression after LNP administration also requires optimization. Because dosing every 2 days limits translational feasibility, future studies could explore the encapsulation of self-amplifying TFEB mRNA in LNPs to prolong expression and reduce dosing frequency.

Collectively, transient liver-targeted TFEB mRNA delivery increased hepatic TFEB expression, altered autophagy-associated and lipid-metabolic programs, and reduced hepatic lipid accumulation in cellular and mouse models of MASLD (Fig. 7g). The data identify FGF21 as a transcriptional target that contributes to the TFEB-associated lipid response. Although durability, optimization, long-term safety, and in vivo pathway dependence remain to be established, these findings support liver-targeted LNP-mRNA delivery as a controllable platform for defining TFEB biology and providing a mechanistic foundation for the future development of precision interventions for metabolic liver disease.

## Materials and methods

### mRNA-LNP preparation

In vitro mRNA synthesis was performed from DNA templates using T7 RNA polymerase. The IVT reaction was prepared as follows: (1) RNase-free water and NTPs were mixed; (2) CleanCap Reagent AG (3′OMe) was added, followed by mixing and centrifugation; (3) 10 × transcription buffer was added to the solution, followed by mixing and centrifugation, and the supernatant was collected; (4) the DNA template was added; (5) mouse RNase inhibitor, yeast inorganic pyrophosphatase, and T7 RNA polymerase were added to the solution, followed by mixing and centrifugation, and the supernatant was collected; (6) after incubation at 37 °C for 2–3 h, DNase I was added to remove the template DNA, and the transcript was purified using the Monarch RNA Cleanup Kit (Thermo Fisher, USA). The mRNA concentration was measured using a NanoDrop spectrophotometer, and mRNA purity was assessed by capillary electrophoresis using a 2100 Bioanalyzer.

LNPs were prepared as follows. The ionizable lipid DLin-MC3-DMA, cholesterol, the helper lipid DSPC, and polyethylene glycol were dissolved in anhydrous ethanol at a molar ratio of 50:38.5:10:1.5 and a total concentration of 10 mg/mL to form the organic phase. mRNA encoding EGFP or TFEB was diluted in sodium citrate buffer (50 mM, pH 4.0) to a final concentration of 0.1 mg/mL to form the aqueous phase. The organic and aqueous phases were mixed at a ratio of 1:3 using a microfluidic device (Inano E, Micro & Nano, Shanghai) at a flow rate of 12 mL/min to obtain the mRNA-LNP mixture. The resulting mRNA-LNP dispersion was diluted with sterile PBS (10 mM, pH 7.2) and transferred to a presterilized Amicon® Ultra-15 centrifugal filter with a molecular weight cutoff of 10 kDa. For buffer exchange and target sample concentration, the samples were centrifuged at 4,000 × g for 15 min at 4 °C, then the concentrate was allowed to equilibrate for 30 min before adding fresh PBS. This procedure was repeated three times to obtain mRNA-LNPs at a final concentration of 2 mg/mL.

### Measurement of LNP particle size and uniformity

The particle size and uniformity of the LNPs were measured using dynamic light scattering (DLS). Concentrated samples were diluted 1:100 in sterile PBS and measured using the HORIBA SZ-100 instrument at 25° and 90° scattering angles. Measurements were performed in triplicate to determine the particle size distribution and polydispersity index (PDI) values of the LNPs. Particle-size distributions were reported on an intensity-weighted basis, and the PDI was used to assess the uniformity and dispersion stability of the LNPs.

### Encapsulation efficiency of mRNA

The mRNA encapsulation efficiency was determined using the Quant-iT™ RiboGreen™ RNA Assay Kit (Invitrogen, R11490). Quant-iT™ RiboGreen® RNA reagent is a highly sensitive fluorescent nucleic acid stain capable of detecting 1–200 ng of nucleic acid in solution. Because the dye does not penetrate intact LNPs, only unencapsulated nucleic acids can be detected before disruption of the particles. Triton X-100 was used as a surfactant to disrupt the LNPs and release the encapsulated mRNA. Treatment with 1% Triton X-100 enabled measurement of the total nucleic acid content. Encapsulation efficiency was calculated from the difference between the nucleic acid amounts measured before and after LNP disruption relative to the total nucleic acid amount, as follows: Encapsulation efficiency (%) = [(amount after disruption − amount before disruption)/amount after disruption] × 100%.

### Animal maintenance and treatments

All animal experiments were conducted in accordance with the guidelines for animal experimentation of the National Institutes of Health, USA, and were approved by the Animal Experiments Ethics Committee of the Animal Center of Southern University of Science and Technology (approval no. SUSTech-JY202302074). C57BL/6 J mice were purchased from Shanghai Model Organisms Center, Inc. (SM-001, Shanghai, China). Mice were housed in an air-conditioned facility under a 12-h light/12-h dark cycle with ad libitum access to water and food. To establish a mouse model of MASLD, 6–8-week-old male mice were fed a choline-deficient, L-amino acid-defined, high-fat diet (CDAHFD; XTMRCD60, Jiangsu Xietong Pharmaceutical Bio-engineering Co., Ltd., Nanjing, China) for 2 weeks. Mice fed a normal chow diet served as controls.

### Cell culture and treatment

Huh-7 cells, a human hepatocellular carcinoma cell line, were purchased from Procell Life Science & Technology Co., Ltd. (CL-0120, Procell, Wuhan, China). Cells were cultured in DMEM supplemented with 10% FBS (10,270–106, Gibco) and 1% penicillin/streptomycin (C0222, Beyotime, Shanghai) at 37 °C in a humidified atmosphere containing 5% CO_2_.

To induce hepatic steatosis in vitro, Huh-7 cells were treated with varying concentrations of palmitic acid (PA; P0500, Sigma, USA) in the culture medium for 24 h. LNP-TFEB and LNP-S211A were added to the culture medium to induce TFEB overexpression in Huh-7 cells, with LNP-EGFP serving as the negative control.

### Quantitative reverse transcription PCR assay

Total RNA from Huh-7 cells was extracted using a Total RNA Extraction Kit (MD021, Magen Biotechnology Co., Ltd., Guangzhou, China). For total RNA extraction from mouse liver tissue, samples were ground in liquid nitrogen and lysed using RNAiso Plus (D9108A, TaKaRa Biotechnology Co., Ltd., Dalian, China) and chloroform. RNA concentration and quality were assessed using a NanoDrop spectrophotometer (A30221, Thermo Fisher Scientific). Subsequently, 1 µg of total RNA from each sample was reverse-transcribed into cDNA using APEXBio reverse transcription reagents in a 20-µL reaction system. The cDNA, SYBR Green Master Mix (B110032, Sangon Biotech, Shanghai, China), qPCR primers, and ddH_2_O were mixed according to the manufacturer’s instructions. qPCR was performed using a Roche LightCycler 480 system (Roche). The relative expression level of each target gene was normalized to 18S and calculated using the 2^ − ΔΔCT^ method. The PCR primer sequences are listed in Table S4.

### Protein extraction and western blotting

Total protein was extracted from Huh-7 cells and mouse liver tissues using RIPA lysis buffer (P0013K, Beyotime, Shanghai, China) supplemented with protease inhibitor (P1008, Beyotime, Shanghai, China), followed by the addition of 5 × loading buffer (P0015L, Beyotime, Shanghai, China). Protein samples were separated by SDS-PAGE and transferred onto nitrocellulose membranes. The membranes were blocked with 5% skim milk for 2 h and then incubated overnight at 4 °C with the following primary antibodies: anti-TFEB (1:1,000, Bethyl, A303-673A), anti-LC3 (1:1,000, Abclonal, A15591), anti-p62 (1:1,000, Abclonal, A7758), anti-phospho-eIF4EBP1 (1:1,000, Abclonal, AP1363), anti-eIF4EBP1 (1:1,000, Abclonal, A24691), anti-FGF21 (1:1,000, Abclonal, A23463), anti-FASN (1:1,000, Epyzime, R013857), anti-SREBP1 (1:1,000, Epyzime, P010134), anti-ACC (1:1,000, Epyzime, P105115), anti-CPT1A (1:1,000, Epyzime, P106431), anti-PPARα (1:1,000, Epyzime, P010554), anti-ATGL (1:1,000, Epyzime, R011456), and anti-β-actin (1:1,000, Proteintech, 20,536–1-AP). After three washes with 1 × TBST, the membranes were incubated with HRP-conjugated anti-rabbit secondary antibody (C31460100, Invitrogen) at room temperature for 45 min. Immunoreactive bands were visualized using an enhanced chemiluminescence substrate, and band intensities were quantified using ImageJ software.

### H&E, Oil Red O, and BODIPY staining

For Oil Red O staining of Huh-7 cells, cells were fixed with 2% paraformaldehyde and stained using an Oil Red O Staining Kit (G1260, Solarbio, Beijing, China). Nuclei were counterstained with hematoxylin. The lipid droplet area was quantified using Image-Pro Plus software (Media Cybernetics, USA) and normalized to the number of cells in each field. For mouse liver tissues, samples were fixed with 4% PFA, dehydrated, and embedded in either OCT compound (Sakura Finetek Co., USA) or paraffin. Frozen sections were cut at a thickness of 8 µm using a cryostat (Leica), whereas paraffin sections were prepared using a microtome (S700A, RWD, Shenzhen, China). After equilibration at room temperature for 2 h, frozen sections were subjected to Oil Red O staining, whereas paraffin sections were used for H&E and immunofluorescence staining. BODIPY staining of frozen sections was performed using BODIPY 558/568 C12 dye (MCE, HY-138226).

### Fluorescence imaging

Following tail-vein injection of varying doses of LNP-EGFP, mouse livers were harvested and processed for paraffin sectioning. Hepatocyte nuclei were counterstained with DAPI. Fluorescence signals were detected using excitation wavelengths of 405 nm for DAPI and 488 nm for EGFP with a Leica DM16000B fluorescence microscope. EGFP fluorescence intensity was quantified using ImageJ and normalized to the number of DAPI-positive cells. For F4/80 immunofluorescence staining, liver sections were blocked with 5% donkey serum for 1 h at room temperature and then incubated overnight at 4 °C with an anti-F4/80 primary antibody (1:100, Cell Signaling Technology, #30325). After washing with PBS, the sections were incubated with an Alexa Fluor-conjugated secondary antibody (1:1,000; Jackson ImmunoResearch Laboratories) for 1 h at room temperature. Immunofluorescence images were acquired using a Leica DM16000B microscope.

### Bulk RNA-sequencing

Sequencing libraries were prepared using the VAHTS Universal V6 RNA-seq Library Prep Kit for Illumina (NR605-02, Vazyme, Nanjing, China). Library preparation included mRNA purification using poly-T beads, mRNA fragmentation, reverse transcription, end repair, addition of a single adenine residue, adapter ligation, and PCR-based amplification and enrichment. Library fragments were purified using the AMPure XP system (Beckman) and quantified using the Agilent High Sensitivity DNA Assay on a Bioanalyzer 2100 system (Agilent). Sequencing was performed on an Illumina NovaSeq X Plus platform. FASTQ files were processed using Salmon. Differential gene expression analysis was performed using the R package DESeq2, followed by enrichment analysis of differentially expressed genes using Metascape.

### Biochemical analyses

Mouse serum was obtained by centrifuging whole-blood samples at 3,000 rpm for 5 min at 20 °C. Serum ALT, AST, and ALP activities and creatinine, cholesterol, and triglyceride concentrations were measured using a fully automated biochemical analyzer (MS480, Medical System Biotechnology Co., Ltd., Ningbo, China). Hepatic triglyceride levels were measured using a triglyceride (TG) content assay kit for liver and adipocyte samples (E1025, Applygen Biotechnology Co., Ltd., Beijing, China).

### Biocompatibility testing

The biocompatibility of the LNP formulations was evaluated using the following assays. (1) CCK-8 assay: 293 A cells were seeded in 96-well plates at 5,000 cells per well and treated with LNP-TFEB or LNP-S211A at 1 μg/mL, with PBS serving as the negative control. CCK-8 reagent (C0038, Beyotime, Shanghai, China) was added to each well, followed by incubation at 37 °C for 2 h. Absorbance at 450 nm (OD450) was measured using a microplate reader. (2) Acute toxicity test in mice: Each mouse received 50 μg of LNP-luciferase, with PBS serving as the negative control. After 24 h, serum was collected for measurement of ALT, AST, ALP, and creatinine levels. (3) In vitro hemolysis assay: Mice were anesthetized, and blood was immediately collected into heparinized centrifuge tubes. The blood samples were diluted with PBS, gently mixed, and centrifuged at 1,500 rpm for 5 min at 4 °C. The erythrocytes were washed with PBS and centrifuged three times. The erythrocyte pellet was then resuspended in PBS to prepare a 5% red blood cell (RBC) suspension. LNP-EGFP, LNP-TFEB, or LNP-S211A was added to the 5% RBC suspension at concentrations of 0.5, 1, or 2 μg/mL. PBS and 0.1% Triton X-100 were used as the negative and positive controls, respectively. After incubation at 37 °C for 2 h, the samples were centrifuged at 2,500 rpm for 5 min, and the absorbance of the supernatant at 540 nm (OD540) was measured using a microplate reader. Hemolysis was calculated as follows: Hemolysis (%) = (OD_(LNP)_—OD_(PBS)_)/(OD_(Triton-X)_—OD_(PBS)_) × 100%.

### In vivo luciferase expression kinetics

LNP-luciferase (10 μg) was administered to mice via tail-vein injection. At 6, 12, 24, and 48 h after injection, luciferin was administered intraperitoneally. Bioluminescence signals were detected using an in vivo small-animal imaging system (IVIS Spectrum, Revvity).

### Chromatin immunoprecipitation assay (ChIP)

Huh-7 cells were transduced with an adenovirus encoding TFEB-FLAG for 48 h. ChIP was performed according to the manufacturer’s protocol (P2078, Beyotime, Shanghai, China). An anti-TFEB antibody (1:500, CST, D2O7D) was used to immunoprecipitate TFEB–DNA complexes, with rabbit control IgG (1:500, Abclonal, AC005) serving as the negative control. qPCR primers targeting the FGF21 promoter were designed based on ChIP-seq data obtained from the GEO database (GSE260617): forward, 5′-AGGGTGAGTCGTCCAAATGA-3′; reverse, 5′-AAACGCTGACATATGCTGCC-3′. Input DNA without immunoprecipitation was used as an internal reference for qPCR quantification.

### siRNA transfection

A total of 100 pmol of siRNA was diluted in 200 μL of OPTI-MEM medium. Subsequently, 8 μL of transfection reagent (40806ES02, YEASEN, Shanghai, China) was added to the siRNA mixture, followed by vortexing for 10 s. The mixture was incubated at room temperature for 10 min and then added to Huh-7 cells cultured in six-well plates. The cells were cultured for an additional 48 h. siRNAs were synthesized by Sangon Biotech. The siRNA sequences were as follows: siFGF21, 5′-CAAUGUUUACCAGUCCGAA-dTdT-3′; siCTL, 5′-UUCUCCGAACGUGUCACGU-dTdT-3′.

### Dual-luciferase reporter assay

The human FGF21 promoter sequence was amplified from HEK293A cell genomic DNA and cloned into a firefly luciferase reporter plasmid to generate the FGF21-promoter construct. A mutant FGF21 promoter reporter plasmid (mutFGF21-promoter) was generated by mutating the predicted CLEAR-box sequence within the FGF21 promoter. HEK293A cells were seeded in 96-well plates and, after reaching confluence, were cotransfected with either the FGF21-promoter or mutFGF21-promoter plasmid together with a Renilla luciferase expression plasmid. After 24 h, the cells were treated separately with LNP-EGFP, LNP-TFEB, or LNP-S211A and incubated for an additional 24 h. Luciferase activity was measured using a microplate reader according to the instructions of the Dual-Lumi™ Luciferase Reporter Gene Assay Kit (RG088S, Beyotime, Shanghai, China). Relative luciferase activity was calculated as the ratio of firefly to Renilla luciferase activity.

### Statistical analysis

Data are presented as mean ± SEM from at least three independent experiments unless otherwise specified in the figure legends. Data normality was assessed before parametric statistical testing. Comparisons between two groups were performed using two-tailed Student’s t-tests, whereas comparisons among multiple groups were performed using one-way analysis of variance (ANOVA) followed by Bonferroni’s post hoc test. Statistical analyses were conducted using GraphPad Prism 10.0, and *P* < 0.05 was considered statistically significant.

## Supplementary Information

Supplementary Material 1. 

Supplementary Material 2. 

## Data Availability

Source data are included in the manuscript and supplementary materials. The RNA-sequencing data have been deposited in the GEO database under accession number GSE283076. Additional data are available from the corresponding author upon reasonable request.
