# Supplementary material for: Lipid nanoparticle-mediated transcription factor EB mRNA delivery for the treatment of metabolic dysfunction-associated steatotic liver disease
Source: Mol Biomed. 2026 Oct 21;7:220. doi: 10.1186/s43556-026-00615-0 (PMC13687531; doi:10.1186/s43556-026-00615-0)
Supplement: Supplementary file 1 [file 43556_2026_615_MOESM1_ESM.docx]

Lipid nanoparticle-mediated transcription factor EB mRNA delivery for the treatment of metabolic dysfunction-associated steatotic liver disease

Wenhao Ju^1,2†^, Chi Zhang^3,4†*^, Tingting Zhang^5†^, Yingjie Liu^3,4^, Xingyun Wang^4,6^, Linjun Zeng^3,4^, Yangpo Cao^3,4^, Haihuan Lin^3,4^, Ouhui Li^3,7^, Peng George Wang^4,6^, Yunjiao He^4,6*^, Haocheng Lu^3,4*^

^1^State Key Laboratory of Cardiovascular Disease，Fuwai Hospital, National Center for Cardiovascular Diseases，Key Laboratory of Innovative Cardiovascular Devices，Chinese Academy of Medical Sciences and Peking Union Medical College, Beijing,100037, China

^2^Yunnan Key Laboratory of Innovative Diagnosis and Treatment of Circulatory Diseases, Fuwai Yunnan Hospital, Chinese Academy of Medical Sciences, Affiliated Cardiovascular Hospital of Kunming Medical University, Kunming, 651012, China

^3^Department of Pharmacology, Joint Laboratory of Guangdong-Hong Kong Universities for Vascular Homeostasis and Diseases, School of Medicine, Southern University of Science and Technology, Shenzhen, Guangdong, 518033, China.

^4^SUSTech Homeostatic Medicine Institute, School of Medicine, Southern University of Science and Technology, Shenzhen 518055, China.

^5^Department of Cardiology, The Eighth Affiliated Hospital, Zhongshan School of Medicine, Sun Yat-sen University, Shenzhen, Guangdong 518033, China

^6^Department of Pharmacology, School of Medicine, Southern University of Science and Technology, Shenzhen, Guangdong, 518033, China.

^7^Key Laboratory of Tropical Translational Medicine of Ministry of Education, Department of Biochemistry and Molecular Biology, School of Basic Medicine and Life Sciences, Haikou, Hainan Medical University

^†^ These authors contributed equally to this work

* Corresponding authors: Chi Zhang, 12331428@mail.sustech.edu.cn; Yunjiao He, [heyj@sustech.edu.cn](mailto:heyj@sustech.edu.cn); Haocheng Lu, [luhc@sustech.edu.cn](mailto:luhc@sustech.edu.cn)

**Supplementary Information**

**Table S1** Differential gene expression by DESeq2, comparing CDAHFD_EGFP vs CHOW_EGFP. baseMean: average of the normalized count values, dividing by size factors, taken over all samples; lfcSE: the standard error estimate for the log2 fold change estimate; stat: Wald statistic; padj: FDR adjusted p value.

**Table S2** Differential gene expression by DESeq2, comparing CDAHFD_TFEB vs CDAHFD_EGFP. baseMean: average of the normalized count values, dividing by size

factors, taken over all samples; lfcSE: the standard error estimate for the log2 fold change estimate; stat: Wald statistic; padj: adjusted p value by FDR.

**Table S3** Differential gene expression by DESeq2, comparing CDAHFD_S211A vs CDAHFD_EGFP. baseMean: average of the normalized count values, dividing by size

factors, taken over all samples; lfcSE: the standard error estimate for the log2 fold change estimate; stat: Wald statistic; padj: adjusted p value by FDR.

**Table S4** A list of primer sequences used for qPCR.

**Fig.S1 LNP-TFEB and S211A did not affect mTORC1 activity.**

Mice were fed a chow or CDAHFD diet continuously for 2 weeks. LNP-EGFP, LNP-TFEB, or LNP-S211A (20 µg) was administered by tail-vein injection every 2 days. mTORC1 activity was determined by phosphorylation of 4EBP1 protein. (n=4)

**Fig.S2 LNP-TFEB and S211A did not cause organ injury.**

(a) H&E staining of the heart, kidneys, lungs, spleen, and inguinal white adipose tissue (iWAT) from mice with MASLD receiving the indicated treatments indicated that LNP-TFEB and S211A did not cause any organ damage. (b) Triglyceride level in mouse inguinal white adipose tissue (iWAT). Scale bar = 500 μm.

**Fig.S3 TFEB improved liver inflammation and fibrosis in MASLD mice.**

The expression of hepatic inflammation-associated genes *Il6*, *IL1β*, and *Tnfa* (a) and fibrosis-related factors *Tgfb1*, *Acta2*, and *Col1a1* (b) in the livers of mice with MASLD was determined by qPCR. (n=6 per group) Data were expressed as mean ± SEM. **P* < 0.05, ***P* < 0.01, ****P* < 0.001, *****P* < 0.0001.

**Fig.S4 TFEB increases FGF21 expression.**

(a) TFEB ChIP-seq data in HeLa and BeWo cells from the GEO database. (b) Putative TFEB-binding CLEAR box in the FGF21 promoter. (c) Huh-7 cells were treated with 10 nM Torin1 or PBS for 24 h and FGF21 expression was detected using Western blot. (n=4 per group) (d) LNP-EGFP, LNP-TFEB, or LNP-S211A were added to cells. After 6 hours, cells were treated with 300μM PA and PBS or the PPARα inhibitor (GW6471, 20 μM) for another 24 hours. Oil Red O staining was used to detect lipid accumulation in Huh-7 cells. Scale bar = 100 μm. (n=6 per group)
